# Randomised trial of chemotherapy versus endocrine therapy in patients presenting with locally advanced breast cancer (a pilot study).

**DOI:** 10.1038/bjc.1991.65

**Published:** 1991-02

**Authors:** J. C. Gazet, H. T. Ford, R. C. Coombes

**Affiliations:** Combined Breast Clinic, St. George's Hospital, London, UK.

## Abstract

Sixty patients with locally advanced breast cancer, but with no evidence of distant metastases were randomised to receive primary endocrine therapy or chemotherapy after assessment and 'Trucut' biopsy of the primary tumour. After 12 weeks all patients were assessed. Eight out of 30 (27%) of the patients who received chemotherapy showed complete clinical regression of the primary cancer, eight patients' tumours had regressed by more than 50%, and ten showed a 25-50% reduction in bi-dimensional diameter. Only four (13%) patients' tumours failed to reduce in size. Seven patients were judged to require mastectomy at the end of the 12 week period of treatment with chemotherapy. In contrast, only three out of 30 (10%) patients receiving endocrine therapy showed a greater than 50% reduction in tumour size, and four patients had a 25-50% reduction at 12 weeks. The remaining patients' tumours either stabilised (12 patients) or enlarged (11 patients). We conclude that primary chemotherapy in patients with primary breast cancer is more effective in rapidly reducing the size of the primary breast cancer than endocrine therapy (P = 0.001) and alters significantly the future management of these patients. However, at 65 weeks on completion of the follow-up, there is no significant difference in the number of patients' disease-free, locally or distant recurrent, or dead.


*Br. J. Cancer* (1991), **63**, 279–282 © Macmillan Press Ltd., 1991

### Randomised trial of chemotherapy versus endocrine therapy in patients presenting with locally advanced breast cancer (a pilot study)

J.-C. Gazet, H.T. Ford & R.C. Coombes

*Combined Breast Clinic, St. George's Hospital, Blackshaw Road, London SW17 0QT, UK.*

**Summary** Sixty patients with locally advanced breast cancer, but with no evidence of distant metastases were randomised to receive primary endocrine therapy or chemotherapy after assessment and 'Trucut' biopsy of the primary tumour. After 12 weeks all patients were assessed. Eight out of 30 (27%) of the patients who received chemotherapy showed complete clinical regression of the primary cancer, eight patients' tumours had regressed by more than 50%, and ten showed a 25–50% reduction in bi-dimensional diameter. Only four (13%) patients' tumours failed to reduce in size. Seven patients were judged to require mastectomy at the end of the 12 week period of treatment with chemotherapy. In contrast, only three out of 30 (10%) patients receiving endocrine therapy showed a greater than 50% reduction in tumour size, and four patients had a 25–50% reduction at 12 weeks. The remaining patients' tumours either stabilised (12 patients) or enlarged (11 patients). We conclude that primary chemotherapy in patients with primary breast cancer is more effective in rapidly reducing the size of the primary breast cancer than endocrine therapy ($P = 0.001$) and alters significantly the future management of these patients. However, at 65 weeks on completion of the follow-up, there is no significant difference in the number of patients' disease-free, locally or distant recurrent, or dead.

It is now generally agreed that conservative surgery by tumour excision is adequate for small cancers (T1 and T2) but the management of patients with locally advanced carcinoma of the breast (T3, T4, N0-2 M0) presents a therapeutic challenge. Local radiation is only partially successful in controlling local tumour growth, with a local relapse rate in 36–72% of patients (Chu *et al.*, 1984; Bruckman *et al.*, 1979; Rubens *et al.*, 1977). Similarly conservative surgery has a similar local relapse rate (Fisher *et al.*, 1985). However, combination therapy of surgery and radiotherapy will control local disease (Veronesi *et al.*, 1986). The problem in the use of these modalities is the high rate of distant metastases uninfluenced by the control of the local disease in the short term.

Primary systemic treatment either with chemotherapy (Delena *et al.*, 1981; Swain *et al.*, 1987; Perloff *et al.*, 1988) or endocrine therapy has been used in the past (Forrest *et al.*, 1986; Gazet *et al.*, 1988). However, no randomised trial has been carried out to examine the relative merits of these two forms of systemic treatment when used in primary treatment of breast cancer in the short term, to influence the secondary treatment offered (surgery or radiotherapy) or in the long term, in the control and reduction of the incidence of metastatic disease.

The objective of this pilot study was 2-fold. First to compare the effectiveness of these two forms of treatment in the short term (12 weeks) and assess the impact of therapy on subsequent treatment and secondly to review the long term effect of therapy at 1 year post primary management (65 weeks).

### Patients and treatment details

Between December 1986 and January 1989, 60 patients, aged 34–69 who presented at the Combined Breast Clinic with locally advanced breast cancer (T3, T4, N0-N2, M0, - UICC TNM Classification (Beahrs & Myers, 1983), were randomised to receive primary treatment with chemotherapy or endocrine therapy. Following preliminary screening by mammography and fine needle aspiration cytology, they were fully staged to exclude metastatic disease. This included full clinical examination, limited skeletal survey, chest X-ray, liver function tests, calcium and full blood count with bone scan and liver ultrasound. All patients had a 'Trucut' biopsy of the breast carcinoma which confirmed the presence of an infiltrating ductal carcinoma in 55 patients and infiltrating lobular carcinoma in five patients.

The patients were fully informed of the object of the trial and written consent obtained in every case according to the Helsinki agreement. The patients were randomised to receive primary treatment with chemotherapy or endocrine therapy over 12 weeks. Patients randomised to receive endocrine therapy were treated with 4-hydroxyandrostenedione 250 mg every 2 weeks by intramuscular injection if postmenopausal (Coombes *et al.*, 1984; Stein *et al.*, 1989), or if premenopausal they received the luteinising hormone releasing hormone analogue goserelin (Zoladex) 3.6 mg subcutaneously every 4 weeks. Those randomised to receive chemotherapy received mitozantrone 7 mg $m^{-2}$, and methotrexate 35 mg $m^{-2}$, 3 weekly four times and mitomycin C 7 mg $m^{-2}$, 6 weekly, intravenously (Powles *et al.*, 1987). There was no significant difference in the comparative age; menopausal status, TNM stage or histology in the two groups (Table I).

All patients were assessed clinically at 4 weekly intervals. Tumour size was measured using calipers and compared with size at presentation. Response was defined by standard UICC criteria (Hayward *et al.*, 1977) i.e. complete response was defined as no palpable mass, partial response as at least a 50% reduction in bidimensional diameter; stable disease either less than a 50% reduction in size or no change. Progressive disease was defined as a $>25\%$ increase in bidimensional diameter. To be considered a response, all measurements had to be confirmed as such in two subsequent separate clinical examinations at 4 weeks apart. At 12 weeks from the start of treatment, the patients were completed restaged and regraded according to the TNM Classification. The treatment policy adopted at this stage depended upon the TNM Classification and the wishes of the patient. Thus if the tumour was considered operable, patients with T1 and T2 tumours were offered wide local excision; those with T3 and T4 tumours, radical mastectomy. If the tumour was inoperable, the patients were offered radical radiotherapy. Where no tumour was clinically palpable, patients were offered radiotherapy as an alternative to radical surgery.

Systemic treatment was only continued post-operatively if the primary tumour either responded or stabilised on systemic therapy: chemotherapy was continued for a further four cycles and endocrine therapy for a further 65 weeks. All patients were staged at the end of the first 12 weeks systemic therapy and/or at the end of the period of treatment.

Correspondence: J.-C. Gazet.
Received 3 April 1990; and in revised form 9 August 1990.

**Table I** Details of patients at induction

| | *Endocrine therapy No (%)* | *Chemotherapy No (%)* |
|---|---|---|
| Mean age (yrs) | 58.9 | 50.8 |
| Range | 42–69 | 34–69 |
| Premenopausal patients | 6 (20) | 10 (30) |
| No with T3 carcinomas (%) | 20 (67) | 19 (63) |
| No with T4 carcinomas (%) | 10 (33) | 11 (37) |
| No with clinically involved nodes | 18 (60) | 14 (47) |
| Histology: infiltrating ductal carcinomas | 29 (97) | 26 (87) |
| Histology: infiltrating lobular carcinomas | 1 (3) | 4 (13) |

### Results of treatment

Table II shows the results of therapy at 12 weeks as assessed by UICC criteria of response and by change of T stage. Chemotherapy resulted in a total of eight complete responders out of 30 patients at 3 months whereas no complete responders were seen in patients receiving endocrine therapy ($P = <0.001$). Eight patients achieved a partial response following chemotherapy and three following endocrine therapy, thus indicating a 53% response rate for chemotherapy with a 10% response rate for endocrine therapy ($P = <0.001$) (Fisher's exact test). A 'minimal' response (between 25–50% reduction) was seen in a further ten patients in the chemotherapy group compared with only four patients in the endocrine therapy.

Using the standard TNM staging, nine out of 30 on endocrine therapy and 19 out of 30 having chemotherapy were downstaged (Table II), ($P = 0.001$). These do not correspond necessarily with the UICC criteria as TNM consider diameter and the UICC percentage reduction in size, but certainly confirm the changes seen.

Table III shows that the definitive treatment at 12 weeks also differed between the two groups. Thus, surgery was carried out in only seven patients in the chemotherapy group whereas this was carried out in 16 patients on the endocrine therapy arm.

We examined side effects in both arms of the study. In the endocrine arm, only 5/30 patients had side effects (4-hydroxyandrostenedione: pain at injection site ($n = 2$); anaphylactoid reaction ($n = 1$); depression ($n = 1$). Goserelin: hot flushes ($n = 1$).

In patients who received chemotherapy 20/30 (67%) patients had side effects. Five patients had both nausea (WHO Grade I) and vomiting and a further four patients had nausea alone, one of which was WHO Grade II. Fourteen patients complained of lethargy, but only five were judged to have WHO GII lethargy. Stomatitis occurred in seven patients, but was WHO GII in only five patients, the remainder being WHO GI. One patient lost her taste and one developed sore eyes. Haematological toxicity was seen in five patients, with the white blood cell count falling to $2.5–3.5 \times 10^9 l^{-1}$ in all five, and one patients's platelet count fell to $30 \times 10^9 l^{-1}$. All these five patients required between 20–30% dose reduction of chemotherapy, three after three cycles and two after five cycles.

None of the patients, however, developed septicaemia or bleeding episodes as a result of treatment.

We did not observe significant changes in the histological appearance using collective histology of the first and second biopsies obtained from the 23 patients who had surgery at 12 weeks.

The results at 65 weeks (Table IV) reveals that at completion of the chemotherapy (eight cycles) or endocrine therapy (78 weeks – reviewed 65 weeks) there is no significant difference in the number who are alive and well in each group. Three patients have died, and three patients have local recurrence in each group, seven have evidence of distant metastases following endocrine therapy compared with only four in the chemotherapy treatment patients. Salvage mastectomy had been performed 32–60 weeks post-presentation in four patients treated with chemotherapy and two patients treated with endocrine therapy.

**Table II** Results of treatment (at 12 weeks)

| *No.* | | *Endocrine therapy 30* | *Chemotherapy 30* | |
|---|---|---|---|---|
| *Response* | | | | |
| Complete response | | 0 | 8 | } $P = 0.001$ |
| Partial response | | 3 | 8 | |
| Minimal response | | 4 | 10 | |
| Stable disease | | 12 | 2 | |
| Progressive disease | | 11 | 2 | |
| Totals | | 30 | 30 | |
| *Response* | *T4 Tumours regraded at 12 weeks | | | |
| 21 | T4 | 6 | 3 | |
| | T3 | 1 | 1 | |
| | T2 | 3 | 4 | |
| | T1 | 0 | 1 | |
| | T0 | 0 | 2 | |
| | *T3 Tumours regraded at 12 weeks | | | |
| 39 | T4 | 3 | 1 | |
| | T3 | 12 | 6 | |
| | T2 | 5 | 4 | |
| | T1 | 0 | 3 | |
| | T0 | 0 | 5 | |
| | | 30 | 30 | |

*Here we have analysed the response of T3 and T4 tumours separately and shown the results of the second assessment at 3 months.

**Table III** Definitive primary treatment (at 12 weeks)

| | *Endocrine therapy* T4 | T3 | *Chemotherapy* T4 | T3 |
|---|---|---|---|---|
| (a) *Surgery* | | | | |
| Radical mastectomy | 0 | 11 | 1 | 4 |
| with radiotherapy | 1 | 1 | 2 | 0 |
| Wide local excision | 0 | 1 | 0 | 0 |
| with radiotherapy | 0 | 2 | 0 | 0 |
| (b) *Radiotherapy* | | | | |
| With endocrine treatment | 6 | 4 | 0 | 0 |
| With chemotherapy | 1 | 0 | 7 | 15 |
| (c) *Chemotherapy** | | | | |
| Sole treatment | 2 | 1 | 1 | 0 |
| Total | 10 | 20 | 11 | 19 |

*These patients had failed to respond to endocrine therapy and two had developed metastatic disease. Chemotherapy was given as palliative therapy.

**Table IV** Result of treatment (65 weeks)

| | *Endocrine therapy* *T4* | *T3* | *Chemotherapy* *T4* | *T3* |
|---|---|---|---|---|
| Dead | 2 | 1 | 1 | 2 (1 LVF) |
| Alive, Distant metastasis | 2 | 5 | 2 | 2 |
| Alive, Local recurrence | 3 | 0 | 1 | 2 |
| Alive and well | 3 | 14 | 7 | 13 |
| Total | 10 | 20 | 11 | 19 |

Survival curves were constructed for patients in each arm of the Study using Cox regression. There was no difference in either relapse-free or overall survival in the two arms.

### Discussion

This is the first reported trial to compare endocrine therapy and chemotherapy as primary treatment for locally advanced breast cancer. The principal finding is that 26/30 (90%) of patients randomised to receive combination chemotherapy showed a greater than 25% reduction in tumour volume at 12 weeks, and in eight patients, the tumour became impalpable. This therapy is well tolerated and resulted in significant myelosuppression in only five patients. Nausea and vomiting was well controlled in all but a minority of patients and alopecia was rarely seen. Twenty-nine of 30 patients completed their chemotherapy. As expected we observed a disappointing response to endocrine therapy as primary therapy, but the side effects were considered minimal. We did not observe the 'flare' occasionally seen in tamoxifen treated patients. The other advantage of 4-hydroxyandrostenedione was that the parenteral route of administration ensured that the patients actually received the treatment. However, only 21 of 30 patients completed their endocrine therapy due to change in treatment because of a change in disease status.

A possible explanation for the response rate is the rate of regression achieved by these different forms of treatment. Thus, endocrine therapy caused a slower reduction in size than chemotherapy, resulting in a response in 7/30 patients. We would expect that certainly a proportion of these patients would achieve partial and/or complete responses if treatment were continued, as we have observed in elderly patients (Gazet *et ai.*, 1988). However, we felt that definitive local treatment in these younger women could not be delayed longer than 12 weeks, and this is reflected in the higher rate of surgery in the endocrine arm of the study. However, at 65 weeks on completion of the trial, it is clear that there has been no significant difference between chemotherapy or endocrine therapy in relation to death, local recurrence, distant metastases and disease-free survival. Secondary surgery is higher in the chemotherapy arm.

Since this study began, we now have clear evidence that immunocytochemical prediction of response to endocrine therapy is reliable (Coombes *et al.*, 1971). Using Trucut biopsy or fine needle aspiration material it is possible to determine the ER status by immunocytochemistry (ERICA) prior to treatment and this allows the preferred treatment, endocrine for ER positive and chemotherapy for ER negative patients, to be offered. In this study, we were able to measure ER by immunotochemistry in 13 patients before treatment and 12/13 showed >50% cells stained i.e. were ER positive (Coombes *et al.*, 1987). Amongst those 12 were the three responders to endocrine therapy. We were able to obtain six samples at 12 weeks for repeat ER and obtained similar results as the pretreatment estimation (both samples positive in five instances and both negative in a single case).

This pilot study has significant limitations in that the small number of patients studied means that we can only analyse response rates in the short term. Future trials will establish the impact of this form of primary systemic treatment on mortality from this disease. However, we feel that improved survival may result since it will be possible to select adjuvant therapy more accurately (a) selecting those that are endocrine sensitive using immunocytochemical tests, (b) by evaluating sensitivity of the primary tumour and thus response of micrometastases (McClelland *et al.*, 1986; Coombes *et al.*, 1986; Mansi *et al.*, 1987).

Our finding has two important clinical implications. Firstly, a significantly greater proportion of patients had conservative surgery or did not require surgery initially because of complete disappearance of the tumour in the chemotherapy arm of the study. Secondly, the degree of reduction in size achieved by primary chemotherapy may well reflect the sensitivity of micrometastases to systemic chemotherapy and this could be a highly significant prognostic feature in patients with breast cancer.

Further studies are now needed to define the role of immunocytochemistry in defining more accurately responders to endocrine therapy. More recently newer techniques which may provide additional information to ER have been adovated including (a) the presence of progesterone receptors that can now be measured immunocytochemically (Perrot-Applanat *et al.*, 1987; Berger *et al.*, 1989); (b) the presence of the oestrogen-induced protein PS2 that is related to outcome (Rio *et al.*, 1988; Skilton *et al.*, 1989) and (c) the absence of EGFR which can also be detected immunocytochemically (Sainsbury *et al.*, 1987).

### References

BEAHRS, O.H. & MYERS, M.H. (eds) (1983). *Manual for staging of cancer* (2nd edition). J.B. Lippincott Co.: Philadelphia, 127.

BERGER, U., WILSON, P., THETHI, S., MCCLELLAND, R.A., GREENE, G.L. & COOMBES, R.C. (1989). Comparison of an immunocytochemical assay for progesterone receptor with a biochemical method of measurement and immunocytochemical examination of the relationship between progesterone and estrogen receptors. *Cancer Res.*, **49**, 5176.

BRUCKMAN, J.E., HARRIS, J.R., LEVENE, M.B., CHAFFEY, J.T. & HELLMAN, S. (1979). Results of treating stage III carcinoma of the breast by primary radiation therapy. *Cancer*, **43**, 985.

CHU, A.M., COPE, O., DOUCETTE, J. & CURRAN, B. (1984). Non-metastatic locally advanced cancer of the breast treated with radiation. *Int. J. Radiat. Oncol. Biol. Phys.*, 10, 2299.

COOMBES, R.C., GOSS, P., DOWSETT, M., GAZET, J.-C. & BRODIE, A. (1984). 4-Hydroxyandrostenedione in treatment of post-menopausal patients with advanced breast cancer. *Lancet*, **ii**, 1237.

COOMBES, R.C., BERGER, U., MANSI, J. & 8 others (1986). Prognostic significance of micrometastases in bone marrow in patients with primary breast cancer. *NCI Monoghraph*, **1**, 51.

COOMBES, R.C., POWLES, T.J., BERGER, U. & 5 others (1987). Prediction of endocrine response in breast cancer by immunocyto-chemical detection of oestrogen receptor in fine needle aspirates. *Lancet*, **ii**, 701.

DELENA, M., VARINI, M., ZUCALI, R. & 5 others (1981). Multimodal treatment for locally advanced breast cancer: results of chemotherapy-radiotherapy versus chemotherapy-surgery. *Cancer Clin. Trials*, 229.

FISHER, B., BAUER, M., MARGOLESE, R. & 16 others (1985). Five-year results of a randomized clinical trial comparing total mastectomy and segmental mastectomy with or without radiation in the treatment of breast cancer. *N. Engl. J. Med.*, **312**, 665.

FORREST, A.P.M., CHETTY, U., MILLER, W.R. & 4 others (1986). A human tumour model. *Lancet*, **ii**, 840.

GAZET, J.-C., MARKOPOULOS, CH., FORD, H.T., COOMBES, R.C., BLAND, J.M. & DIXON, R.C. (1988). Prospective randomised trial of tamoxifen versus surgery in elderly patients with breast cancer. *Lancet*, **i**, 679.

HAYWARD, J.L., CARBONNE, P.P., HEUSON, J.-C., KUMAOKA, S., SEGALOFF, A. & RUBENS, R.D. (1977). Assessment of response to therapy in advanced breast cancer. *Cancer*, **39**, 1289.

MANSI, J.L., BERGER, U., EASTON, D. & 6 others (1987). Micrometastases in bone marrow in patients with primary breast cancer: evaluation as an early predictor of bone metastases. *Br. Med. J.*, **295**, 1093.

MCCLELLAND, R.A., BERGER, U., MILLER, L.S., POWLES, T.J. & COOMBES, R.C. (1986). Immunocytochemical assay for oestrogen receptor in patients with breast cancer: relationship to a biochemical assay and to outcome of therapy. *J. Clin. Oncol.*, **4**, 1171.

PERLOFF, M., LESNICK, G.J., KORZUN, A. & 9 others (1988). Combination chemotherapy (CAFVP) with mastectomy or radiotherapy for stage III breast carcinoma: a CALGB study. *J. Clin. Oncol.*, **6**, 261.

PERROT-APPLANAT, M., GROYER-PICARD, M.-T., LORENZO, F. & 5 others (1987). Immunocytochemical study with monoclonal antibodies to progesterone receptor in human breast tumours. *Cancer Res.*, **47**, 2652.

POWLES, T.J., ASHLEY, S.E., FORGESON, G.V. & 4 others (1987). Treatment of advanced breast cancer with mitomycin-C, mitoxantrone and methotrexate (3M) compared to vincristine, anthra-cycline and cylophosphamide (VAC). In *Clinical Progress with Mitoxantrone*. Bonadonna, G. (ed.) London, Royal Society of Medicine Services Limited. Series No. 110, pp. 1–7.

RIO, M.C., BELLOCQ, J.P., GAIRARD, B. & 7 others (1988). Specific expression of the pS2 gene in subclasses of breast cancers in comparison with expression of the estrogen and progesterone receptors and oncogene ERB-B2. *Proc. Natl Acad. Sci. USA*, **84**, 9243.

RUBENS, R.D., ARMITAGE, P., WINTER, P.J., TONG, D. & HAYWARD, J.L. (1977). Prognosis in inoperable stage III carcinoma of the breast. *Eur. J. Cancer*, **13**, 805.

STEIN, R.C., DOWSETT, M., HEDLEY, A. & 4 others (1990). Treatment of advanced breast cancer in post-menopausal women with 4-hydroxyandrostenedione. *Cancer Chemother. & Pharmacol.*, **26**, 75.

SAINSBURY, J.R.C., FARNDON, J.R., NEEDHAM, G.K., MALCOLM, A.J. & HARRIS, A.L. (1987). Epidermal-Growth-Factor receptor status as predicator of early recurrence of and death from breast cancer. *Lancet*, **i**, 1398.

SKILTON, R.A., LUQMANI, Y.A., MCCLELLAND, R.A. & COOMBES, R.C. (1989). Characterisation of a messenger RNA selectively expressed in human breast cancer. *Br. J. Cancer*, **60**, 168.

SWAIN, S., SORACE, R.A., BAGLEY, C.S. & 5 others (1987). Neo-adjuvant chemotherapy in the combined modality approach of locally advanced non-metastatic breast cancer. *Cancer Res.*, **47**, 3889.

VERONESI, U., BANFI, A., DEL VECCHIO, M. & 11 others (1986). Comparison of Halsted mastectomy with quadrantectomy, axillary dissection, and radiotherapy in early breast cancer: long-term results. *Eur. J. Cancer Clin. Oncol.*, **22**, 1085.


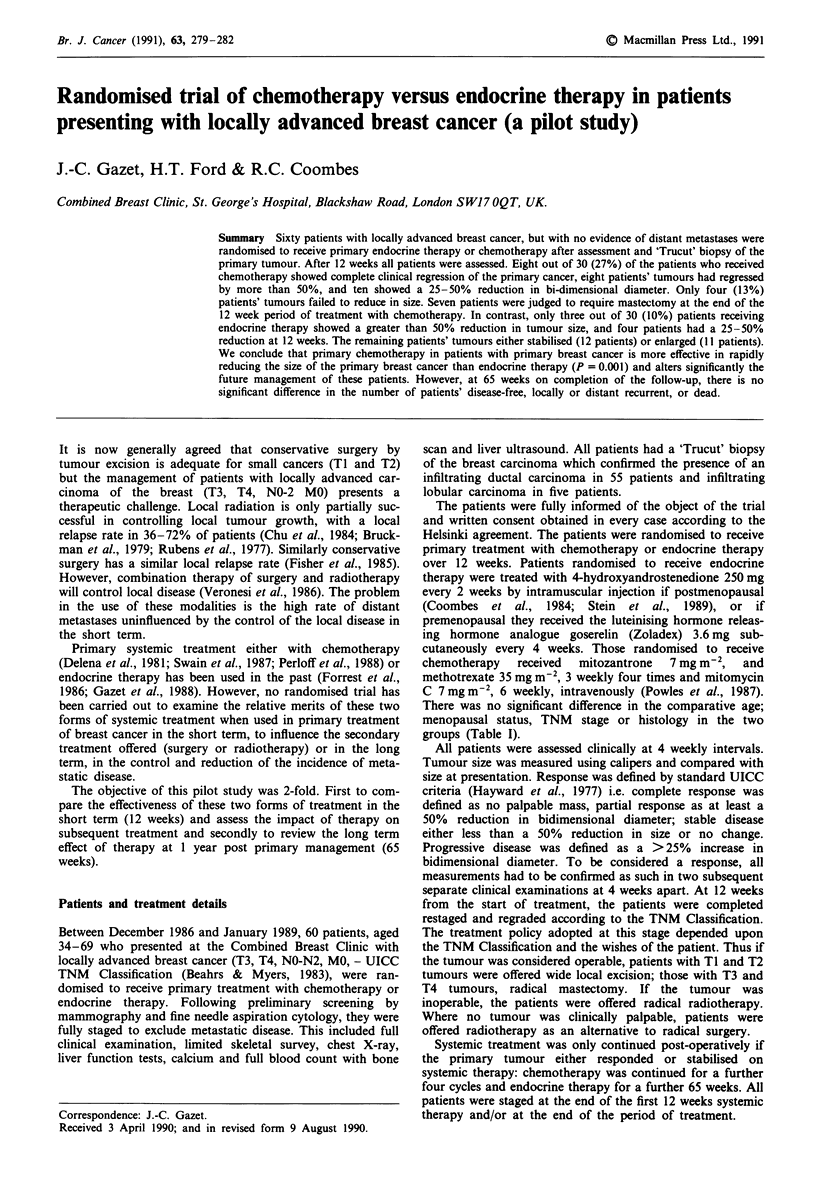

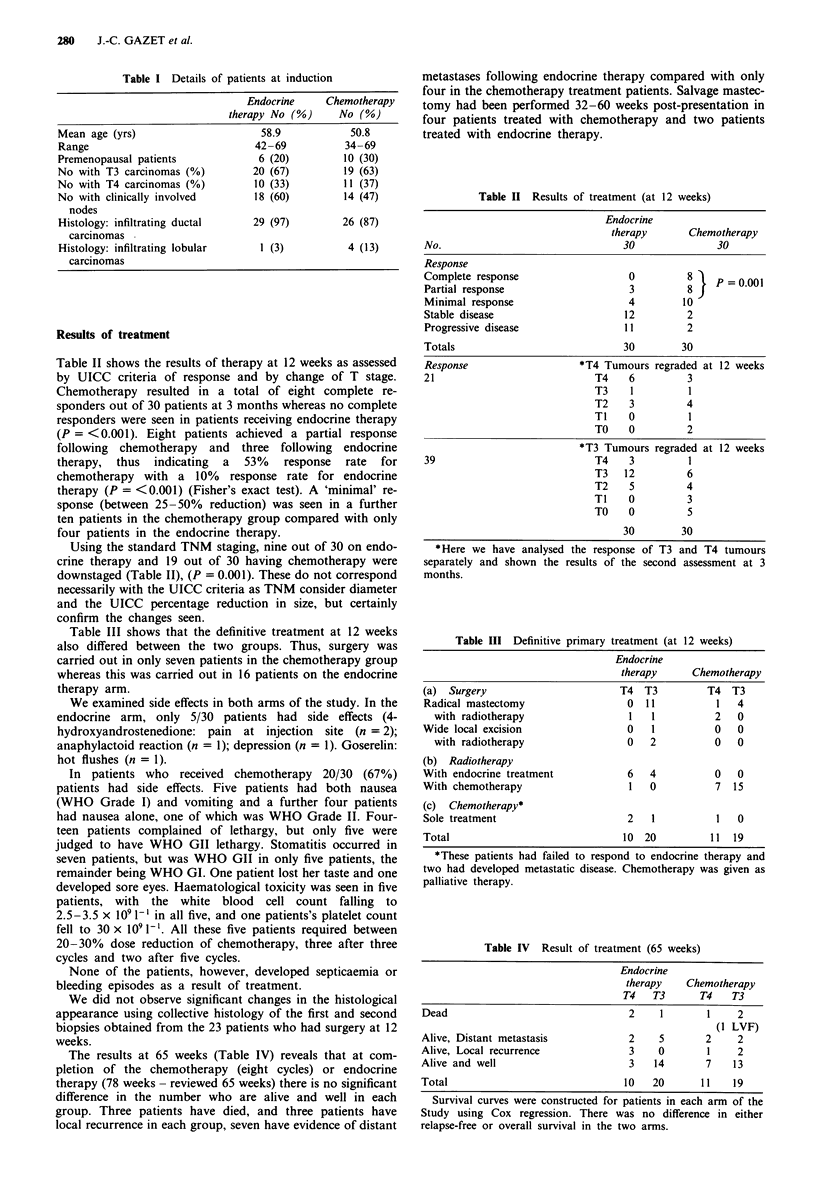

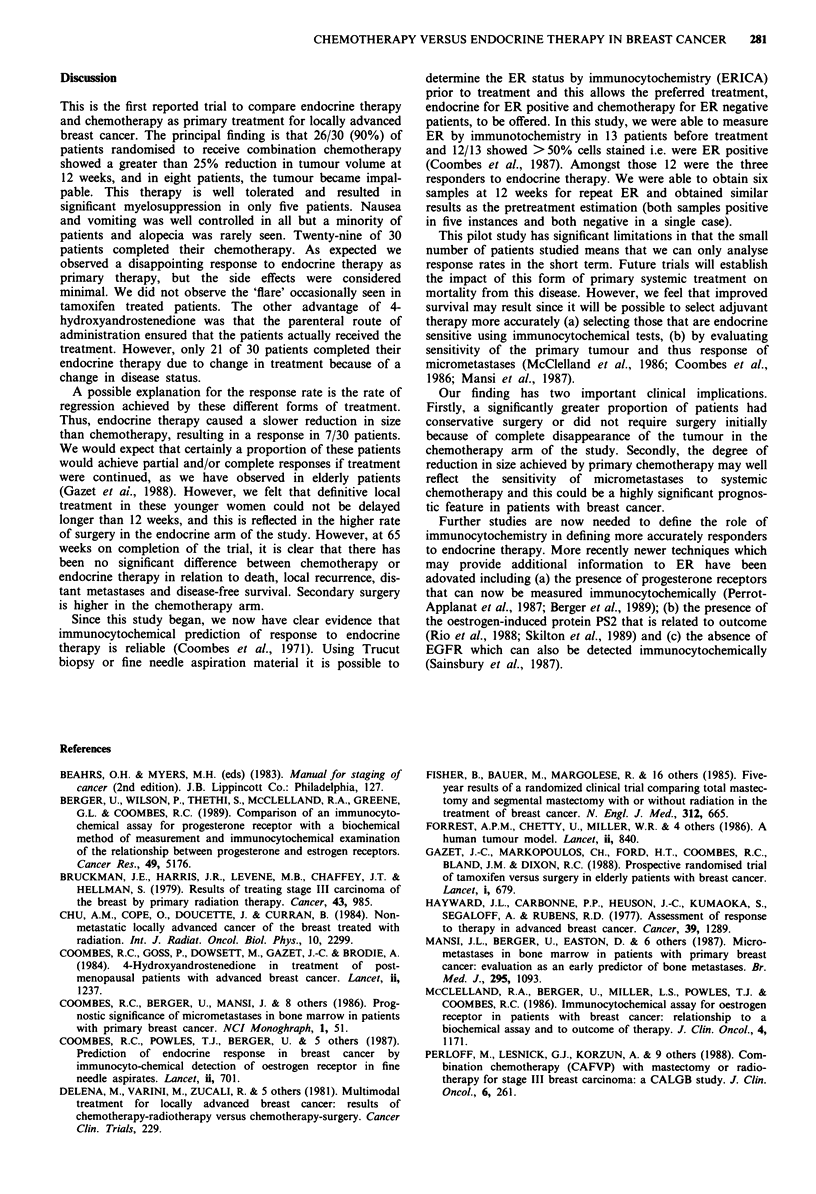

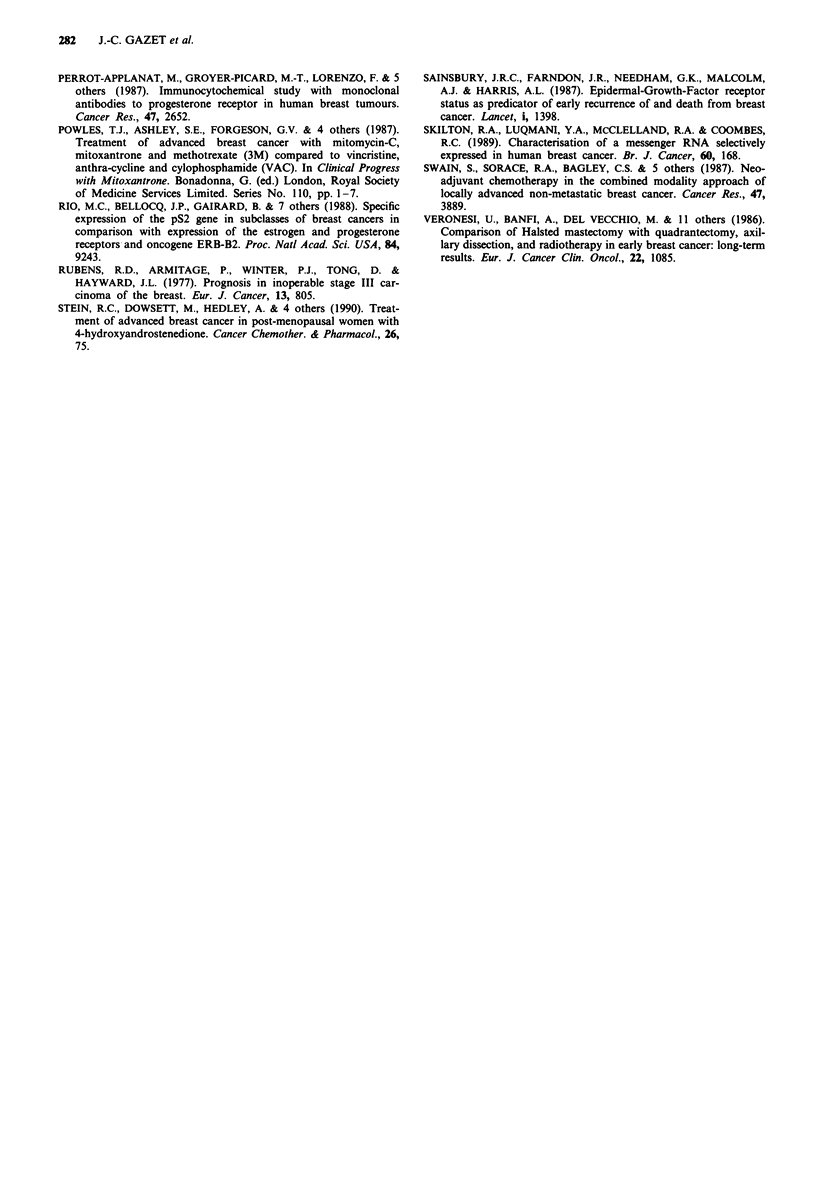


## References

[OCR_00405] Berger U., Wilson P., Thethi S., McClelland R. A., Greene G. L., Coombes R. C. (1989). Comparison of an immunocytochemical assay for progesterone receptor with a biochemical method of measurement and immunocytochemical examination of the relationship between progesterone and estrogen receptors.. Cancer Res.

[OCR_00413] Bruckman J. E., Harris J. R., Levene M. B., Chaffey J. T., Hellman S. (1979). Results of treating stage III carcinoma of the breast by primary radiation therapy.. Cancer.

[OCR_00418] Chu A. M., Cope O., Doucette J., Curran B. (1984). Non-metastatic locally advanced cancer of the breast treated with radiation.. Int J Radiat Oncol Biol Phys.

[OCR_00423] Coombes R. C., Goss P., Dowsett M., Gazet J. C., Brodie A. (1984). 4-Hydroxyandrostenedione in treatment of postmenopausal patients with advanced breast cancer.. Lancet.

[OCR_00436] Coombes R. C., Powles T. J., Berger U., Wilson P., McClelland R. A., Gazet J. C., Trott P. A., Ford H. T. (1987). Prediction of endocrine response in breast cancer by immunocytochemical detection of oestrogen receptor in fine-needle aspirates.. Lancet.

[OCR_00440] De Lena M., Varini M., Zucali R., Rovini D., Viganotti G., Valagussa P., Veronesi U., Bonadonna G. (1981). Multimodal treatment for locally advanced breast cancer. Result of chemotherapy-radiotherapy versus chemotherapy-surgery.. Cancer Clin Trials.

[OCR_00446] Fisher B., Bauer M., Margolese R., Poisson R., Pilch Y., Redmond C., Fisher E., Wolmark N., Deutsch M., Montague E. (1985). Five-year results of a randomized clinical trial comparing total mastectomy and segmental mastectomy with or without radiation in the treatment of breast cancer.. N Engl J Med.

[OCR_00452] Forrest A. P., Levack P. A., Chetty U., Hawkins R. A., Miller W. R., Smyth J. F., Anderson T. J. (1986). A human tumour model.. Lancet.

[OCR_00458] Gazet J. C., Markopoulos C., Ford H. T., Coombes R. C., Bland J. M., Dixon R. C. (1988). Prospective randomised trial of tamoxifen versus surgery in elderly patients with breast cancer.. Lancet.

[OCR_00462] Hayward J. L., Carbone P. P., Heuson J. C., Kumaoka S., Segaloff A., Rubens R. D. (1977). Assessment of response to therapy in advanced breast cancer: a project of the Programme on Clinical Oncology of the International Union Against Cancer, Geneva, Switzerland.. Cancer.

[OCR_00467] Mansi J. L., Berger U., Easton D., McDonnell T., Redding W. H., Gazet J. C., McKinna A., Powles T. J., Coombes R. C. (1987). Micrometastases in bone marrow in patients with primary breast cancer: evaluation as an early predictor of bone metastases.. Br Med J (Clin Res Ed).

[OCR_00473] McClelland R. A., Berger U., Miller L. S., Powles T. J., Coombes R. C. (1986). Immunocytochemical assay for estrogen receptor in patients with breast cancer: relationship to a biochemical assay and to outcome of therapy.. J Clin Oncol.

[OCR_00480] Perloff M., Lesnick G. J., Korzun A., Chu F., Holland J. F., Thirlwell M. P., Ellison R. R., Carey R. W., Leone L., Weinberg V. (1988). Combination chemotherapy with mastectomy or radiotherapy for stage III breast carcinoma: a Cancer and Leukemia Group B study.. J Clin Oncol.

[OCR_00488] Perrot-Applanat M., Groyer-Picard M. T., Lorenzo F., Jolivet A., Vu Hai M. T., Pallud C., Spyratos F., Milgrom E. (1987). Immunocytochemical study with monoclonal antibodies to progesterone receptor in human breast tumors.. Cancer Res.

[OCR_00502] Rio M. C., Bellocq J. P., Gairard B., Rasmussen U. B., Krust A., Koehl C., Calderoli H., Schiff V., Renaud R., Chambon P. (1987). Specific expression of the pS2 gene in subclasses of breast cancers in comparison with expression of the estrogen and progesterone receptors and the oncogene ERBB2.. Proc Natl Acad Sci U S A.

[OCR_00509] Rubens R. D., Armitage P., Winter P. J., Tong D., Hayward J. L. (1977). Prognosis in inoperable stage III carcinoma of the breast.. Eur J Cancer.

[OCR_00520] Sainsbury J. R., Farndon J. R., Needham G. K., Malcolm A. J., Harris A. L. (1987). Epidermal-growth-factor receptor status as predictor of early recurrence of and death from breast cancer.. Lancet.

[OCR_00526] Skilton R. A., Luqmani Y. A., McClelland R. A., Coombes R. C. (1989). Characterisation of a messenger RNA selectively expressed in human breast cancer.. Br J Cancer.

[OCR_00531] Swain S. M., Sorace R. A., Bagley C. S., Danforth D. N., Bader J., Wesley M. N., Steinberg S. M., Lippman M. E. (1987). Neoadjuvant chemotherapy in the combined modality approach of locally advanced nonmetastatic breast cancer.. Cancer Res.

[OCR_00537] Veronesi U., Banfi A., Del Vecchio M., Saccozzi R., Clemente C., Greco M., Luini A., Marubini E., Muscolino G., Rilke F. (1986). Comparison of Halsted mastectomy with quadrantectomy, axillary dissection, and radiotherapy in early breast cancer: long-term results.. Eur J Cancer Clin Oncol.

